# Economic instability and substance use among adults in 10 US states: Evidence from a population-based study

**DOI:** 10.1371/journal.pone.0360066

**Published:** 2026-10-07

**Authors:** Peiyuan Liu, Tania de Jesús Espinosa, Elham Samami, Bernard Mensah, Hermine Poghosyan

**Affiliations:** 1 School of Nursing, Yale University, Orange, Connecticut, United States of America; 2 COPPER Center, Yale School of Medicine, New Haven, Connecticut, United States of America; NYU Grossman School of Medicine: New York University School of Medicine, UNITED STATES OF AMERICA

## Abstract

**Background:**

Economic instability may shape substance use (i.e., cigarette use and cannabis use) among the adult population. However, their relationship remains poorly understood. This study estimated the prevalence of economic instability indicators (i.e., employment instability, received food stamps, food insecurity, housing insecurity, and threatened utility shutoffs), exclusive cigarette use (TU), exclusive cannabis use (CU), and cigarette-cannabis co-use (TCU), and investigated the associations between economic instability indicators and these distinct substance use patterns among adults aged ≥18 years.

**Methods:**

This study used cross-sectional population-based data from the 2023 Behavioral Risk Factor Surveillance System (BRFSS) Marijuana Use and Social Determinants and Health Equity modules, administered in 10 US states. The 2023 BRFSS achieved a response rate of 44.7%. The sample included (unweighted n = 50,481; weighted N = 24,896,969) adults ≥18 years. The outcome variables were TU, CU, and TCU. Indicators of economic instability included employment instability, receipt of food stamps, food insecurity, housing insecurity, and threats of utility shutoffs. Weighted descriptive statistics and multivariable multinomial logistic regression analyses were conducted.

**Results:**

Overall, 48.6% of the participants were aged ≤50 years, 52.6% were women, and 64.4% self-identified as non-Hispanic White. The prevalences of TU, CU, and TCU were 8.1%, 11.1%, and 3.6%, respectively. Participants experiencing employment instability, housing insecurity, or threats of utility shutoffs were more likely to engage in CU compared to TU or TCU (*P* ≤ 0.01). All economic instability indicators were significantly associated with TCU, with the strongest association observed for those who received food stamps (aRRR = 2.27, 95% CI: 1.75–2.94), which was also significantly associated with TU (aRRR = 1.55, 95% CI: 1.24–1.93). Threats of utility shutoffs (aRRR = 1.37, 95% CI: 1.10–1.70) and housing insecurity (aRRR = 1.27, 95% CI: 1.04–1.55) were significantly associated with CU.

**Conclusions:**

Economic instability indicators are significantly associated with cigarette and cannabis use, as well as their co-use patterns among US adults residing in the 10 participating states. Effective, targeted public health interventions to mitigate substance use risk in economically precarious populations are needed. Healthcare providers should routinely screen for economic instability to identify individuals at high risk for substance use and provide them with social services and behavioral health interventions.

## Introduction

In the United States, cigarette use has declined to historic lows, while cannabis use has steadily increased. In fact, adult cigarette use prevalence dropped from 42.0% in 1965 to just 9.6% in 2022 [1,2]. In contrast, cannabis use has surged; nearly 19% of Americans, about 52.5 million people, reported using cannabis at least once in 2021 [3]. This rise coincided with the expansion of state-level cannabis legalization, with both medical and recreational use policies associated with increased cannabis use among adults [4,5]. According to the 2023 National Survey on Drug Use and Health, 15.4% of the US population aged 12 years or older used cannabis in the previous month, surpassing the proportion who smoked cigarettes [6].

Amid these shifts, growing concern surrounds the co-use of tobacco and cannabis products, which poses unique and significant health risks. Specifically, individuals who use both products, tobacco and cannabis, are exposed to significantly higher levels of toxicants, with biomarker studies showing carcinogen levels up to 464% higher than those who use cigarettes alone [7]. Co-use can also reinforce dependence on both nicotine and tetrahydrocannabinol (THC), the primary psychoactive component of cannabis, which may hinder cessation efforts for both substances [8,9]. Further, the co-use can complicate smoking cessation efforts and increase the risk of prolonged use and associated health harms [10]. For example, about 23.0% of individuals who reported co-use of both products were able to quit cigarette use after 7 months, compared with 28.9% of those reporting no use [10]. Taken together, these findings indicate that co-use of tobacco and cannabis products compounds health risks, deepens dependence, and undermines efforts to quit either substance.

Substance use behaviors cannot be understood solely in biomedical terms. The stress-coping framework posits that chronic stress drives individuals toward substances such as nicotine or cannabis as maladaptive coping strategies [11]. Socially rooted stressors, including food insecurity [12], housing instability [13], unemployment [14], and broader economic hardships, heighten vulnerability to substance use and addiction [11]. Individuals facing socioeconomic hardships consistently demonstrate higher rates of cigarette and drug use [11], a burden further compounded by the disproportionate concentration of cannabis retailers in low-income and racially marginalized communities following legalization [15], underscoring the important role of social determinants of health. Increasingly, research frames substance use not simply as a matter of personal choice but as an outcome of structural inequities [16,17], pointing to the need for interventions that alleviate stressors and strengthen coping resources in low-income communities [11]. Despite the recognized links between economic hardships and health behaviors, few studies have examined how it shapes patterns of cigarette use, cannabis use, and their co-use at the population level. To address this gap, the present study aims to: 1) estimate the prevalence of economic instability indicators (i.e., employment instability, received food stamps, food insecurity, housing insecurity and threatened utility shutoffs), 2) estimate the prevalence of exclusive traditional cigarette use (TU), exclusive cannabis use (CU), and cigarette-cannabis co-use (TCU); and 3) investigate the associations between economic instability indicators and these distinct substance use patterns among adults aged ≥18 years.

## Methods

### Data source and study population

The current study used data from the 2023 Behavioral Risk Factor Surveillance System (BRFSS), incorporating both the Marijuana Use and the Social Determinants and Health Equity (SDHE) optional modules [18]. BRFSS is a nationally representative cross-sectional survey that collects information from non-institutionalized adults aged ≥18 years, living in the US. The survey is administered annually and includes three components: a core set of questions asked by all states, optional modules that states may choose to include based on relevance to specific public health topics, and state-added questions tailored to local priorities. It collects data on demographics, health-related behaviors, chronic health conditions, access to care, and other health indicators. The 2023 BRFSS surveyed a total of 433,323 individuals, achieving a response rate of 44.7% [18].

We used data from 10 US states (Connecticut, Delaware, Illinois, Indiana, Maine, Mississippi, Montana, Nevada, New Mexico, and Virginia) that administered both the Marijuana Use and SDHE modules as part of the 2023 BRFSS survey [18]. Adults aged ≥18 years living in selected states were included. After excluding states that did not administer both modules and observations that had responses as “refused”, “don’t know/not sure”, or had missing values in study variables, the final analytic sample included 50,481 adults aged ≥18 years. S1 Fig shows details about the sample selection (Supplement).

### Ethics approval statement

BRFSS data were publicly available and de-identified; thus, the current study did not require institutional review board approval, as it met the exception criteria outlined in 45 CFR part 46.102 [19].

### Measures

#### Outcome variable.

The outcome variables were exclusive traditional cigarette use (TU), exclusive cannabis use (CU), and the cigarette-cannabis co-use (TCU). To assess traditional cigarette use, participants were initially asked, “Have you smoked at least 100 cigarettes in your entire life?” Those who reported “yes” were then asked the following question, “Do you now smoke cigarettes every day, some days, or not at all?”. Using these two items, individuals were categorized into two groups: 1) participants who report current cigarette use (those who had smoked at least 100 cigarettes in their lifetime and currently smoked either every day or some days). 2) never/formerly used cigarettes (those who either had never used 100 cigarettes in their lifetime or had smoked 100 cigarettes but no longer smoke) [20]. CU was determined through the following survey item: “During the past 30 days, on how many days did you use marijuana or cannabis?” with response options of “none” and “1-30”. Respondents who reported using cannabis at least one day during the past 30 days were categorized as currently using cannabis [21]. We combined TU and CU into a single variable with the following categories: 0 = no substance use; 1 = exclusive TU; 2 = exclusive CU; and 3 = TCU.

#### Key independent variable.

Economic instability indicators were the key independent variables. They were assessed using five items from the SDHE module. These items were designed to capture multiple aspects of economic instability experienced by respondents over the past 12 months. These included: 1) employment instability, whether the respondents had lost employment or experienced reduced working hours; 2) received food stamps through supplemental nutrition assistance program (SNAP); 3) food insecurity, frequency of the purchased food that did not last, and there were no money to get more; 4) housing insecurity, unable to pay mortgage, rent or utility bills; and 5) experiences of threatened utility shutoffs of electric, gas, oil, or water company threatened to shut off services [22]. S1 Table describes each item in detail (Supplement).

#### Covariates.

To account for potential confounding factors in the relationship between economic instability indicators and substance use, we included a robust set of covariates encompassing demographic, socio-economic, and health factors. Demographic variables included age (18–49, 50–64, 65–74, 75–79, ≥ 80), sex (male, female), race and ethnicity (non-Hispanic White, non-Hispanic Black, Hispanic, other), and marital status (never married, married, divorced or separated, widowed). Socioeconomic variables included education attainment (high school graduate or less, attended college or technical school, college degree), metropolitan residency status (yes, no), and health insurance coverage (yes, no). Health-related variables included general health status (fair or poor, good, very good, or excellent), stress, and chronic health conditions. Perceived stress was assessed by the question “Within the last 30 days, how often have you felt this kind of stress?” We categorized responses as yes (usually, always) or no (sometimes, never, rarely). Chronic health conditions included the history of cardiovascular disease (i.e., myocardial infarction, stroke, angina), lung disease (i.e., asthma, chronic obstructive pulmonary disease, emphysema, or chronic bronchitis), and cancer history (non-skin cancer). These covariates were selected a *priori* based on clinical relevance and established factors that influence the association with economic instability and substance use to control for potential confounding [12,14,23].

### Statistical analysis

All analyses accounted for the complex survey design of the BRFSS by applying appropriate sampling weights using the ‘svy’ command in STATA 18 (StataCorp LLC, College Station, TX) [24]. We computed descriptive statistics to characterize the sample, reported unweighted frequencies and weighted percentages to provide population-level estimates. Bivariate analyses were conducted to examine associations between economic instability indicators and substance use. We conducted five separate multivariable multinomial logistic regression models to examine the relationship between each of the five economic instability indicators and substance use, with non-users serving as the reference group and adjusting for a robust set of covariates. We reported results using relative risk ratio (RRR) with 95% confidence interval (CI). Results from regression models were visualized using forest plots. Multicollinearity was checked before regression analysis. A statistical significance threshold of *P* ≤ 0.05 was used to determine significant associations.

## Results

### Study sample characteristics

Table 1 summarizes the characteristics of the study participants. Roughly half (48.6%) of the participants were aged <50 years, and 52.6% were women. Approximately 64.4% of participants self-identified as non-Hispanic White, 57.3% were married, and 32.7% had a college degree. Among participants, 79.6% resided in metropolitan areas, 92.8% had health insurance coverage, 48.3% reported their general health status as good, and 13.2% experienced stress in the past 30 days. Overall, the prevalence rates of lung disease, cardiovascular disease, and cancer history was 19.3%, 9.0%, and 8.8%, respectively.

**Table 1:** Characteristics of study participantsparticipants.

Characteristics	Unweighted sample n = 50,481	Weighted prevalence (95% CI), N = 24,896,969
Age, years		
18-49	16,198	48.6 (47.8, 49.5)
50-64	13,308	26.0 (25.3, 26.8)
65-74	11,962	15.3 (14.7, 15.8)
75-79	4,407	4.9 (4.6, 5.2)
≥80	4,606	5.2 (4.9, 5.6)
Sex		
Female	27,463	52.6 (51.7, 53.4)
Male	23,018	47.4 (46.6, 48.3)
Race and ethnicity		
Non-Hispanic White	39,563	64.4 (63.5, 65.2)
Non-Hispanic Black	4,104	13.2 (12.6, 13.8)
Hispanic	3,806	13.8 (13.2, 14.4)
Other	3,008	8.6 (8.1, 9.2)
Marital status		
Never married	8,118	22.8 (22.0, 23.6)
Married	28,775	57.3 (56.4, 58.1)
Divorced or separated	7,732	12.3 (11.8, 12.8)
Widowed	5,856	7.6 (7.2, 8.1)
Education attainment		
High school graduate or less	14,549	36.9 (36.1, 37.8)
Attended college or technical school	13,204	30.4 (29.5, 31.2)
College degree	22,728	32.7 (32.0, 33.5)
Metropolitan residency status		
No	16,574	20.4 (19.9, 21.0)
Yes	33,907	79.6 (79.0, 80.1)
Health insurance coverage		
No	2,441	7.2 (6.7, 7.7)
Yes	48,040	92.8 (92.4, 93.3)
General health status		
Fair or poor	9,428	18.6 (17.9, 19.3)
Good	24,557	48.3 (47.5, 49.2)
Very good or excellent	16,496	33.1 (32.3, 33.9)
Perceived stress		
No	44,748	86.8 (86.1, 87.3)
Yes	5,733	13.2 (12.7, 13.9)
Lung disease history		
No	40,217	80.7 (80.0, 81.4)
Yes	10,264	19.3 (18.6, 20.0)
Cardiovascular disease history		
No	44,565	91.0 (90.5, 91.5)
Yes	5,916	9.0 (8.5, 9.5)
Cancer history		
No	44,081	91.1 (90.7, 91.6)
Yes	6,400	8.9 (8.4, 9.3)

### Substance use and economic instability

Table 2 shows the prevalence of economic instability indicators and substance use among study participants. In total, 12.0% reported employment instability, 10.4% obtained food stamps, 13.9% faced food insecurity, 11.6% experienced housing insecurity, and 7.7% reported threats of utility shutoffs. Among participants, 8.1% reported exclusive TU, 11.1% exclusive CU, and 3.6% reported TCU (Table 2).

**Table 2:** Characteristics of substance use and economic instability indicators.

Characteristics	Unweighted sample, n = 50,481	Weighted prevalence (95% CI), N = 24,896,969
Employment instability		
No	46,210	88.0 (87.4, 88.7)
Yes	4,271	12.0 (11.3, 12.6)
Received food stamps		
No	45,968	89.6 (89.0, 90.1)
Yes	4,513	10.4 (9.9, 11.0)
Food insecurity		
No	45,071	86.1 (85.5, 86.7)
Yes	5,410	13.9 (13.3, 14.5)
Housing insecurity		
No	46,113	88.4 (87.8, 89.0)
Yes	4,368	11.6 (11.0, 12.2)
Threatened utility shutoffs		
No	47,276	92.3 (91.8, 92.8)
Yes	3,205	7.7 (7.2, 8.2)
Cigarette cannabis co-use		
None	39,672	77.1 (76.4, 77.9)
TU	3,943	8.1 (7.6, 8.7)
CU	5,150	11.1 (10.6, 11.7)
TCU	1,716	3.6 (3.3, 4.0)

Table 3 illustrates sample characteristics by economic instability indicators. There were statistically significant differences in sample characteristics by economic instability indicators. Participants with employment instability were predominantly aged 18–49 years (71.2%, *P* ≤ 0.01), non-Hispanic White (45.9%), had a high school education or less (48.4%), and lived in metropolitan areas (83.2%). Notably, those receiving food stamps were primarily never married (37.4%), while marriage was more common among participants with other indicators of economic instability. Women were more likely than men to report food insecurity (60.0% vs 40.0%, *P* ≤ 0.01) and housing insecurity (62.0% vs. 38.0%, *P* ≤ 0.01). Participants who reported employment instability, housing insecurity, and utility shutoffs were more likely to report CU than to report TU or TCU (*P* ≤ 0.01). Participants who reported receiving food stamps and experienced food insecurity were more likely to report TU than CU or TCU (*P* ≤ 0.01).

**Table 3:** Characteristics of participants by economic instability, weighted.

Characteristics	Economic instability, % (%95 CI)*				
	Employment instability	Received food stamp	Food insecurity	Housing insecurity	Threatened utility shutoffs
Age, years	< 0.01	< 0.01	< 0.01	< 0.01	< 0.01
18-49	71.2 (68.5, 73.7)	61.9 (59.1, 64.6)	63.0 (60.6, 65.3)	69.1 (66.5, 71.7)	67.0 (63.7, 70.1)
50-64	22.6 (20.3, 25.1)	24.9 (22.4, 27.6)	23.4 (21.4, 25.5)	22.6 (20.4, 25.1)	24.3 (21.4, 27.5)
65-74	5.6 (4.5, 7.0)	9.4 (8.1, 10.9)	8.8 (7.7, 10.0)	5.4 (4.6, 6.5)	6.2 (5.2, 7.4)
75-79	0.4 (0.3, 0.6)	2.4 (1.9, 3.1)	2.4 (1.9, 2.9)	1.1 (0.7, 1.6)	1.2 (0.7, 1.8)
≥80	0.3 (0.1, 0.5)	1.4 (1.1, 1.8)	2.5 (1.8, 3.6)	1.7 (0.9, 3.2)	1.4 (0.8, 2.2)
Sex	0.27	< 0.01	< 0.01	< 0.01	< 0.01
Female	51.0 (48.1, 53.9)	67.8 (65.1, 70.4)	60.0 (57.6, 62.4)	62.0 (59.2, 64.6)	60.4 (57.0, 63.6)
Male	49.0 (46.1, 51.9)	32.2 (29.6, 34.9)	40.0 (37.6, 42.4)	38.0 (35.4, 40.8)	39.6 (36.4, 43.0)
Race and ethnicity	< 0.01	< 0.01	< 0.01	< 0.01	< 0.01
Non-Hispanic White	45.9 (43.1, 48.8)	42.9 (40.2, 45.7)	40.6 (38.3, 42.9)	39.8 (37.1, 42.4)	44.3 (41.1, 47.6)
Non-Hispanic Black	19.2 (17.0, 21.7)	26.2 (23.7, 28.9)	21.8 (19.8, 24.0)	22.3 (19.9, 24.9)	24.7 (21.8, 27.9)
Hispanic	25.7 (23.2, 28.4)	20.3 (17.9, 22.9)	27.9 (25.6, 30.3)	27.8 (25.3, 30.5)	20.9 (18.0, 24.2)
Other	9.2 (7.7, 10.9)	10.6 (8.8, 12.7)	9.7 (8.2, 11.4)	10.2 (8.4, 12.2)	10.0 (8.0, 12.6)
Marital status	< 0.01	< 0.01	< 0.01	< 0.01	< 0.01
Never married	40.0 (37.2, 43.0)	37.4 (34.5, 40.3)	32.5 (30.2, 34.9)	32.7 (30.0, 35.4)	31.2 (28.1, 34.4)
Married	42.1 (39.4, 45.0)	30.9 (28.2, 33.6)	39.0 (36.6, 41.4)	39.9 (37.1, 42.7)	39.9 (36.6, 43.2)
Divorced or separated	14.5 (12.9, 16.3)	23.9 (21.8, 26.1)	21.1 (19.3, 23.1)	21.1 (19.2, 23.2)	22.4 (20.0, 25.1)
Widowed	3.3 (2.0, 5.3)	7.9 (6.2, 10.0)	7.4 (6.0, 9.0)	6.3 (4.7, 8.4)	6.5 (4.4, 9.5)
Education attainment	< 0.01	< 0.01	< 0.01	< 0.01	< 0.01
High school or less	48.4 (45.5, 51.3)	58.3 (55.4, 61.2)	60.4 (57.9, 62.8)	55.8 (53.0, 58.6)	50.8 (47.4, 54.2)
Attended college or technical school	33.6 (30.8, 36.6)	31.6 (28.8, 34.4)	29.9 (27.6, 32.3)	32.1 (29.5, 34.8)	34.6 (31.3, 38.1)
College degree	18.0 (16.3, 19.8)	10.2 (8.7, 11.9)	9.7 (8.5, 11.1)	12.1 (10.6, 13.9)	14.6 (12.8, 16.7)
Metropolitan residency	< 0.01	< 0.01	< 0.01	0.72	0.27
No	16.8 (15.0, 18.7)	24.2 (22.1, 26.4)	22.9 (21.1, 24.8)	20.1 (18.2, 22.2)	21.7 (19.4, 24.2)
Yes	83.2 (81.3, 85.0)	75.8 (73.6, 77.9)	77.1 (75.2, 78.9)	79.9 (77.8, 81.8)	78.3 (75.8, 80.6)
Health insurance	< 0.01	0.30	< 0.01	< 0.01	< 0.01
No	19.3 (17.3, 21.6)	6.5 (5.4, 7.9)	17.0 (15.1, 18.9)	17.9 (15.9, 20.1)	15.3 (13.1, 17.8)
Yes	80.7 (78.4, 82.7)	93.5 (92.1,94.6)	83.0 (81.1, 84.9)	82.1 (79.9, 84.1)	84.7 (82.2, 86.9)
General health status	< 0.01	< 0.01	< 0.01	< 0.01	< 0.01
Fair or poor	25.1 (22.8, 27.5)	37.1 (34.4, 39.8)	37.7 (35.4, 40.1)	36.3 (33.7, 39.0)	35.4 (32.3, 38.6)
Good	38.2 (35.4, 41.0)	27.4 (25.0, 30.1)	26.3 (24.1, 28.5)	27.1 (24.7, 29.6)	28.2 (25.3, 31.3)
Very good or excellent	36.8 (33.9, 39.7)	35.5 (32.6, 38.4)	36.0 (33.6, 38.6)	36.6 (33.8, 39.5)	36.4 (33.1, 39.9)
Stress	< 0.01	< 0.01	< 0.01	< 0.01	< 0.01
No	76.4 (74.0, 78.6)	71.0 (68.2, 73.6)	69.0 (66.7, 71.3)	63.7 (60.9, 66.5)	62.5 (59.2, 65.8)
Yes	23.6 (21.4, 26.0)	29.0 (26.4, 31.8)	31.0 (28.7, 33.3)	36.3 (33.5, 39.1)	37.5 (34.2, 40.8)
Lung disease history	< 0.01	< 0.01	< 0.01	< 0.01	< 0.01
No	76.3 (73.8, 78.7)	68.0 (65.2, 70.6)	70.8 (68.5, 73.0)	70.0 (67.4, 72.6)	68.8 (65.7, 71.8)
Yes	23.7 (21.3, 26.2)	32.0 (29.4, 34.8)	29.2 (27.0, 31.5)	30.0 (27.4, 32.6)	31.2 (28.2, 34.3)
Cardiovascular disease history	< 0.01	< 0.01	< 0.01	0.11	0.13
No	94.4 (93.1, 95.5)	88.1 (86.3, 89.8)	87.4 (85.7, 88.9)	89.8 (88.1, 91.3)	89.7 (87.7, 91.3)
Yes	5.6 (4.5, 6.9)	11.9 (10.2, 13.7)	12.6 (11.1, 14.3)	10.2 (8.7, 11.9)	10.3 (8.7, 12.3)
Cancer history	< 0.01	0.16	0.28	0.04	0.13
No	94.4 (92.3, 95.9)	92.5 (90.2, 94.2)	92.0 (90.0, 93.6)	92.9 (90.8, 94.5)	92.9 (90.0, 95.0)
Yes	5.6 (4.1, 7.7)	7.6 (5.8, 9.8)	8.0 (6.4, 10.0)	7.1 (5.5, 9.2)	7.1 (5.0, 10.0)
Substance use	< 0.01	< 0.01	< 0.01	< 0.01	< 0.01
None	66.9 (64.1, 69.6)	62.1 (59.1, 64.9)	63.5 (61.1, 65.9)	62.0 (59.2, 64.8)	58.5 (55.0, 61.8)
TU	9.4 (7.7, 11.4)	14.1 (12.1, 16.5)	14.0 (12.3, 15.9)	12.9 (11.0, 15.1)	14.8 (12.2, 17.9)
CU	16.2 (14.3, 18.4)	13.7 (11.7, 16.0)	13.6 (11.9, 15.4)	15.4 (13.5, 17.6)	16.1 (13.8, 18.7)
TCU	7.5 (6.1, 9.2)	10.1 (8.4, 12.1)	8.9 (7.6, 10.4)	9.7 (8.1, 11.5)	10.6 (8.7, 12.9)

* Differences between groups were compared by the chi-square test.

### Association between economic instability indicators and substance use

In the unadjusted regression models, all indicators of economic instability were significantly associated with substance use. These associations remained largely consistent in the adjusted multivariable logistic regression models. Detailed results from regression models on the relationship between economic instability indicators and each substance outcomes are provided below and illustrated in Fig 1. Additionally, tables presenting results from regression models are included in the Supplement (S2 Table).

**Fig 1:**
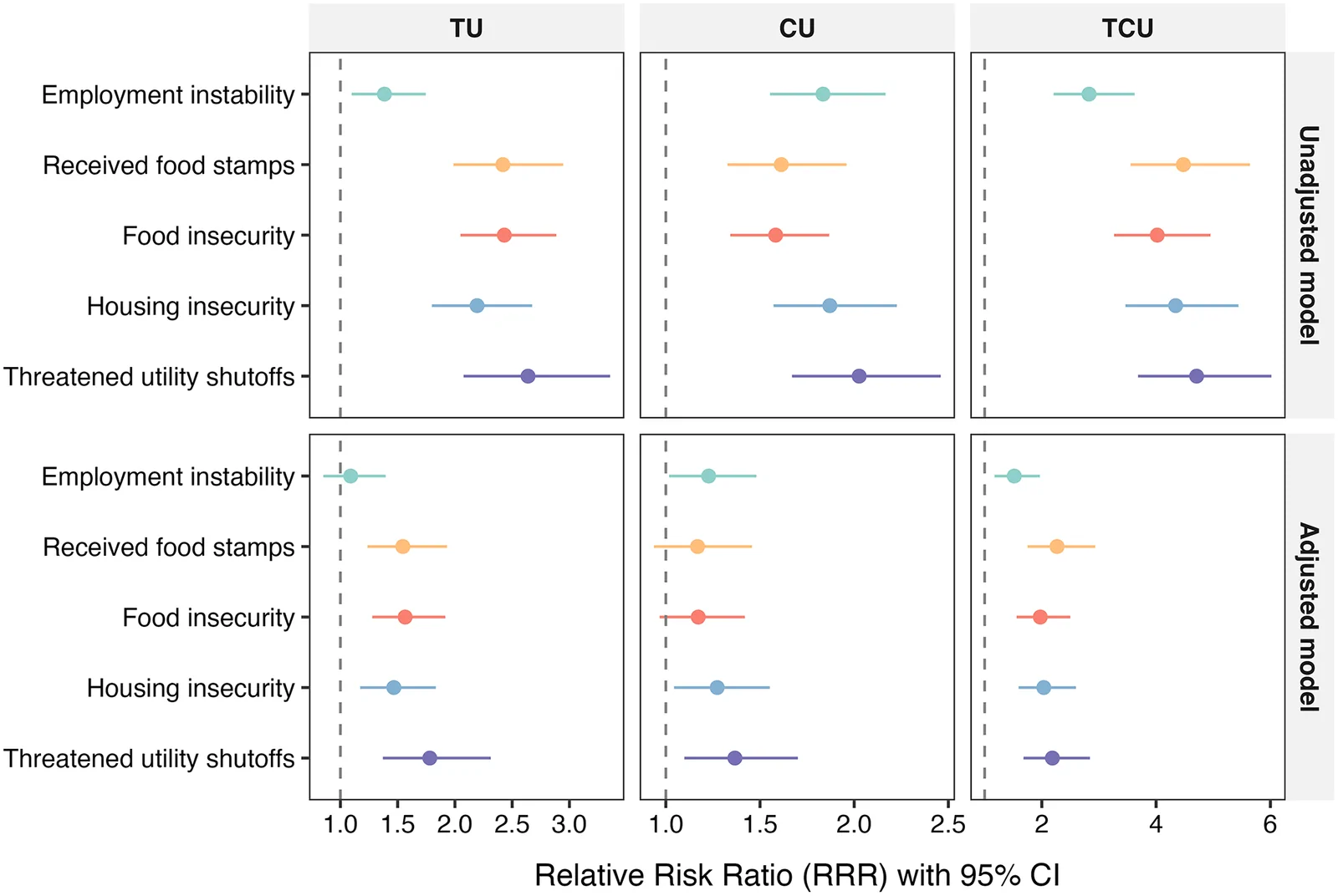
Association between economic instability and substance use. TU, exclusive traditional cigarette use; CU, exclusive cannabis use; TCU, cigarette-cannabis co-use. RRR, relative risk ratio. CI, confidence interval. Adjusted models accounted for age, sex, race, marital status, education attainment, metropolitan residency status, health insurance coverage, general health status, stress, lung disease history, cardiovascular disease history, and cancer history.

### Economic instability indicators and cigarette-cannabis co-use

Participants who received food stamps had the highest relative risk of TCU, being 2.27 times more likely to report TCU than those not receiving food stamps (aRRR = 2.27; 95% CI:1.75–2.94). This was followed by individuals who experienced threatened utility shutoffs, who were 2.19 times more likely to report TCU than those who did not experience such an event (aRRR = 2.19; 95% CI: 1.68–2.84). Similarly, participants reporting housing insecurity (aRRR = 2.03; 95% CI: 1.59–2.60), food insecurity (aRRR = 1.97; 95% CI: 1.56–2.50), and employment instability (aRRR = 1.52; 95% CI: 1.17–1.97) also showed increased likelihood of TCU relative to those without such economic instability.

### Economic instability indicators and exclusive cannabis use

Significant associations were identified between employment instability (aRRR = 1.23; 95% CI: 1.02–1.48), housing insecurity (aRRR = 1.27; 95% CI:1.04–1.55), threatened utility shutoffs (aRRR = 1.37; 95% CI: 1.10–1.70), and exclusive CU. The relationship between food insecurity and receipt of food stamps and exclusive CU was not statistically significant.

### Economic instability indicators and exclusive traditional cigarette use

Participants who received food stamps (aRRR = 1.55; 95% CI 1.24–1.93), reported food insecurity (aRRR = 1.57; 95% CI: 1.28–1.92), housing insecurity (aRRR = 1.47; 95% CI: 1.17–1.83), and threatened utility shutoffs (aRRR = 1.78; 95% CI:1.37–2.31) had an increased likelihood of reporting exclusive TU compared to their counterparts. The relationship between employment instability and exclusive TU was not statistically significant.

## Discussion

Using a large, multi-state population-based dataset of US adults from 10 states, our study findings show that economic instability indicators are statistically significantly associated with patterns of cigarette and cannabis use. Notably, distinct types of economic instability are associated with specific substance use patterns, with economic instability indicators showing stronger associations with TCU than with exclusive TU and CU. The strongest association was found between the receipt of SNAP benefits (food stamps), housing insecurity, threatened utility shutoffs, and TCU. Individuals in these groups were more than twice as likely to engage in TCU compared to their economically stable counterparts. This aligns with prior research showing that acute financial shocks, such as threatened utility shutoffs and job loss, exacerbate stress and increase susceptibility to substance use [14,25,26]. This magnitude of risk, significantly higher than for exclusive use of either substance, suggests that co-use may serve as a cumulative response to compounding stressors, where chronic deprivation (need for food assistance) intersects with acute crises (threat of utility loss). These findings underscore the need for integrated prevention strategies that address economic instability alongside substance use.

Study findings also showed that food insecurity is primarily linked to exclusive TU, while housing insecurity and employment issues are associated with exclusive CU. Consistent with established research linking poverty to cigarette use, we found that food-related hardship was a dominant correlate [23]. Specifically, participants who reported receiving SNAP and those experiencing food insecurity were approximately 1.5 times more likely to smoke cigarettes exclusively. However, the observed association should be interpreted cautiously, as SNAP receipt may reflect not only economic hardship but also successful access to supportive social services. Unlike CU, employment instability was not a statistically significant factor for exclusive TU. Together, these findings suggest that substance use prevention and intervention strategies may be more effective when they are tailored to address specific forms of economic instability rather than economic hardship broadly.

In our multi-state sample, 11.1% reported exclusive CU, surpassing the 8.1% reporting exclusive TU, and 3.6% reporting TCU. These findings show that CU is now more prevalent than cigarette use among US adults residing in the 10 participating states, reflecting national trends of declining cigarette use and increasing CU [1,27]. In the current literature, reported TCU rates have been mixed, likely due to differences in study methodology, populations, and the timeframe of the datasets used, making direct comparisons difficult. A recent study reported comparable findings, with 3.7% of participants using cannabis alongside cigarettes or electronic cigarettes [8]. However, direct comparison is limited because that study was conducted among adults aged ≥50 years, whereas our study sample included a broader range of adults aged ≥18 years. Another study reported that 9.3% of young adults engaged in TCU [28], while Sedani and colleagues reported substantially higher rates of about 36% of participants who reported current cigarette use also used cannabis [29]. These mixed findings, with wide variation in reported TCU prevalence, underscore the need for future research and standardized reporting to understand patterns of TCU among US adults better.

Our study findings showed that economic instability indicators, including employment instability, receipt of food stamps, food insecurity, housing insecurity, and threatened utility shutoffs, were prevalent among the US adult population from 10 states. The most prevalent form of economic instability was food insecurity, reported by 13.9% of individuals, underscoring the challenges many face in meeting essential needs. This finding is consistent with national data indicating that 13.5% of US households were food insecure at some time during 2023 [30].

Addressing substance use requires more than just changing individual behavior. It also involves tackling the structural and material contexts that shape those behaviors. Routine screening for food, housing, and utility insecurity in clinical and community settings, along with co-located cessation and support programs within safety-net services (e.g., SNAP or housing assistance), may effectively reach high-risk populations. Policies that stabilize household finances, such as preventing utility shutoffs and expanding income supports, could indirectly reduce substance use by easing chronic stress and economic strain.

### Limitations

There are several limitations to be acknowledged in this study. First, the cross-sectional design of this study prevents establishing causality between economic instability and substance use. Longitudinal cohort studies are needed to establish the temporal relationship. Second, the data analyzed here were derived from 10 US states that administered both the Marijuana Use and SDHE modules, which is not fully nationally representative. BRFSS data were collected via telephone and achieved a response rate of 44.7%, which suggests a potential non-response rate, particularly among economically marginalized or hard-to-reach populations. Hence, future studies should incorporate a broader geographic scope and more inclusive data collection methods to improve generalizability. Third, since BRFSS relies on self-reported data, there is a risk of recall bias or misclassification. Despite controlling for covariates, some potential confounders were not controlled, such as family history of substance use disorders and history of trauma. Fourth, the measure of economic instability may also not fully capture the experiences of hardship, particularly in terms of severity and persistence. Fifth, substance use was not measured in detail, lacking information on duration, frequency, intensity, and methods of use. Sixth, we did not examine effect modification by demographic characteristics such as age and race/ethnicity, which are critical for targeted intervention development. These gaps present valuable opportunities for future research to comprehensively assess the relationship between economic instability indicators and substance use, thereby clarifying the underlying mechanisms and informing the development of targeted interventions and policies.

## Conclusion

Economic instability indicators are strongly associated with cigarette use, cannabis use, and especially their co-use. National and state-level strategies that promote economic security, affordable housing, and utility stability can reduce such stressors that perpetuate substance use. Integrating economic screening tools into primary care and behavioral health systems would allow providers to identify individuals at risk early and connect them to social resources. Cross-sector collaborations between public health, housing, and social welfare agencies are needed to align resource delivery and expand access to supports such as food assistance, rental relief, and income stabilization programs. By integrating economic and behavioral health strategies, policymakers can reduce or prevent substance use and promote population health.

## Supporting information

S1 Fig Flow Chart displaying sample selection. (TIF)

S1 Table Variables to measure economic stability. (PDF)

S2 Table Association between economic stability and substance use. (PDF)
