# Supplementary material for: Economic instability and substance use among adults in 10 US states: Evidence from a population-based study
Source: PLoS One. 2026 Oct 7;21(10):e0360066. doi: 10.1371/journal.pone.0360066 (PMC13645042; doi:10.1371/journal.pone.0360066)
Supplement: Supplementary file 1 [file pone.0360066.s002.pdf]

S1 Table. Variables to measure economic stability.

Variable names	Questions asked with options	Categories
Employment instability	In the past 12 months have you lost employment or had hours reduced?	0=No<br>1=yes
Received food stamp	During the past 12 months, have you received food stamps, also called SNAP, the Supplemental Nutrition Assistance Program on an EBT card?	0=no<br>1=yes
Food insecurity	During the past 12 months how often did the food that you bought not last, and you did not have money to get more? Was that…	0 =no (rarely-never)<br>1=yes (always-usually-sometimes)
Housing insecurity	During the past 12 months, was there a time when you were not able to pay your mortgage, rent or utility bills?	0=no<br>1=yes
Threatened utility shutoffs	During the past 12 months, was there a time when an electric, gas, oil, or water company threatened to shut off services?	0=No<br>1=yes
