# Supplementary material for: Economic instability and substance use among adults in 10 US states: Evidence from a population-based study
Source: PLoS One. 2026 Oct 7;21(10):e0360066. doi: 10.1371/journal.pone.0360066 (PMC13645042; doi:10.1371/journal.pone.0360066)
Supplement: Supplementary file 2 [file pone.0360066.s003.pdf]

S2 Table. Association between economic stability and substance use.

Model	Variable	Outcome	RRR	95% CI
Unadjusted	Employment instability	TU	1.38	(1.10, 1.74)
	Employment instability	CU	1.83	(1.55, 2.17)
	Employment instability	TCU	2.83	(2.20, 3.62)
	Received food stamps	TU	2.42	(1.99, 2.95)
	Received food stamps	CU	1.61	(1.33, 1.96)
	Received food stamps	TCU	4.48	(3.55, 5.64)
	Food insecurity	TU	2.43	(2.05, 2.89)
	Food insecurity	CU	1.58	(1.34, 1.87)
	Food insecurity	TCU	4.02	(3.26, 4.95)
	Housing insecurity	TU	2.19	(1.80, 2.67)
	Housing insecurity	CU	1.87	(1.57, 2.23)
	Housing insecurity	TCU	4.34	(3.46, 5.44)
	Threatened utility shutoffs	TU	2.64	(2.08, 3.35)
	Threatened utility shutoffs	CU	2.03	(1.67, 2.46)
	Threatened utility shutoffs	TCU	4.71	(3.68, 6.02)
Adjusted*	Employment instability	TU	1.09	(0.85, 1.40)
	Employment instability	CU	1.23	(1.02, 1.48)
	Employment instability	TCU	1.52	(1.17, 1.97)
	Received food stamps	TU	1.55	(1.24, 1.93)
	Received food stamps	CU	1.17	(0.94, 1.46)
	Received food stamps	TCU	2.27	(1.75, 2.94)
	Food insecurity	TU	1.57	(1.28, 1.92)
	Food insecurity	CU	1.17	(0.97, 1.42)
	Food insecurity	TCU	1.97	(1.56, 2.50)
	Housing insecurity	TU	1.47	(1.17, 1.83)
	Housing insecurity	CU	1.27	(1.04, 1.55)
	Housing insecurity	TCU	2.03	(1.59, 2.60)
	Threatened utility shutoffs	TU	1.78	(1.37, 2.31)
	Threatened utility shutoffs	CU	1.37	(1.10, 1.70)
	Threatened utility shutoffs	TCU	2.19	(1.68, 2.84)

* TU, exclusive traditional cigarette use; CU, exclusive cannabis use; TCU, cigarette-cannabis co-use. RRR, relative risk ratio. CI, confidence interval. Adjusted models accounted for age, sex, race, marital status, education attainment, metropolitan residency status, health insurance coverage, general health status, stress, lung disease history, cardiovascular disease history, and cancer history.
